# Learning from data to predict future symptoms of oncology patients

**DOI:** 10.1371/journal.pone.0208808

**Published:** 2018-12-31

**Authors:** Nikolaos Papachristou, Daniel Puschmann, Payam Barnaghi, Bruce Cooper, Xiao Hu, Roma Maguire, Kathi Apostolidis, Yvette P. Conley, Marilyn Hammer, Stylianos Katsaragakis, Kord M. Kober, Jon D. Levine, Lisa McCann, Elisabeth Patiraki, Eileen P. Furlong, Patricia A. Fox, Steven M. Paul, Emma Ream, Fay Wright, Christine Miaskowski

**Affiliations:** 1 Centre for Vision, Speech and Signal Processing, University of Surrey, Guildford, United Kingdom; 2 University of California, San Francisco, United States of America; 3 University of Strathclyde, Glasgow, Scotland; 4 European Cancer Patient Coalition, Brussels, Belgium; 5 School of Nursing, University of Pittsburgh, Pittsburgh, United States of America; 6 Department of Nursing, Mount Sinai Medical Center, New York, United States of America; 7 Faculty of Nursing, University of Peloponnese, Sparti, Greece; 8 National and Kapodistrian University of Athens, Athens, Greece; 9 UCD School of Nursing, Midwifery and Health Systems, Dublin, Ireland; 10 School of Nursing, Yale University, New Haven, United States of America; Zapadoceska univerzita, CZECH REPUBLIC

## Abstract

Effective symptom management is a critical component of cancer treatment. Computational tools that predict the course and severity of these symptoms have the potential to assist oncology clinicians to personalize the patient’s treatment regimen more efficiently and provide more aggressive and timely interventions. Three common and inter-related symptoms in cancer patients are depression, anxiety, and sleep disturbance. In this paper, we elaborate on the efficiency of Support Vector Regression (SVR) and Non-linear Canonical Correlation Analysis by Neural Networks (n-CCA) to predict the severity of the aforementioned symptoms between two different time points during a cycle of chemotherapy (CTX). Our results demonstrate that these two methods produced equivalent results for all three symptoms. These types of predictive models can be used to identify high risk patients, educate patients about their symptom experience, and improve the timing of pre-emptive and personalized symptom management interventions.

## Introduction

A growing body of evidence, [1–3] as well as clinical experience suggests that the symptom experience of oncology patients is extremely variable. While some patients experience very few symptoms, other patients undergoing the same treatment experience multiple co-occurring symptoms that are severe and extremely distressing. The clinical dilemma is how to identify these high risk patients prior to the initiation of treatment, so that aggressive symptom management interventions can be initiated and deleterious outcomes can be avoided. The application of machine learning techniques to develop algorithms to identify this high risk phenotype is the first step toward individualized symptom management.

For this investigation, we applied machine learning techniques to develop an algorithm that could identify patients with the highest severity scores for three common and inter-related symptoms (i.e., depression, anxiety, sleep disturbance). Depression occurs in up to 60% of cancer patients. [4] Between 35% and 53% of patients report anxiety during cancer treatment [5] and 45% of patients experience both of these symptoms. [6] Equally deleterious and linked to both depression and anxiety are complaints of sleep disturbance in 30% to 50% of oncology patients. [7] All three symptoms are associated with decrements in patients’ ability to function on a daily basis as well as on their quality of life. Of note, according to a systematic review by Alvaro et al. [8] as depression, anxiety and sleep disturbances are often grouped together, the treatment of insomnia may prevent the development of anxiety and depressive disorders, and vice-versa. Therefore, if we can predict the patients who are at a higher risk for these symptoms, treatments can be initiated to manage these symptoms. In addition, these efficient machine learning methods could be used to predict the severity other symptom in patients with cancer, as well as in patients with other chronic medical conditions.

**Fig 1:**
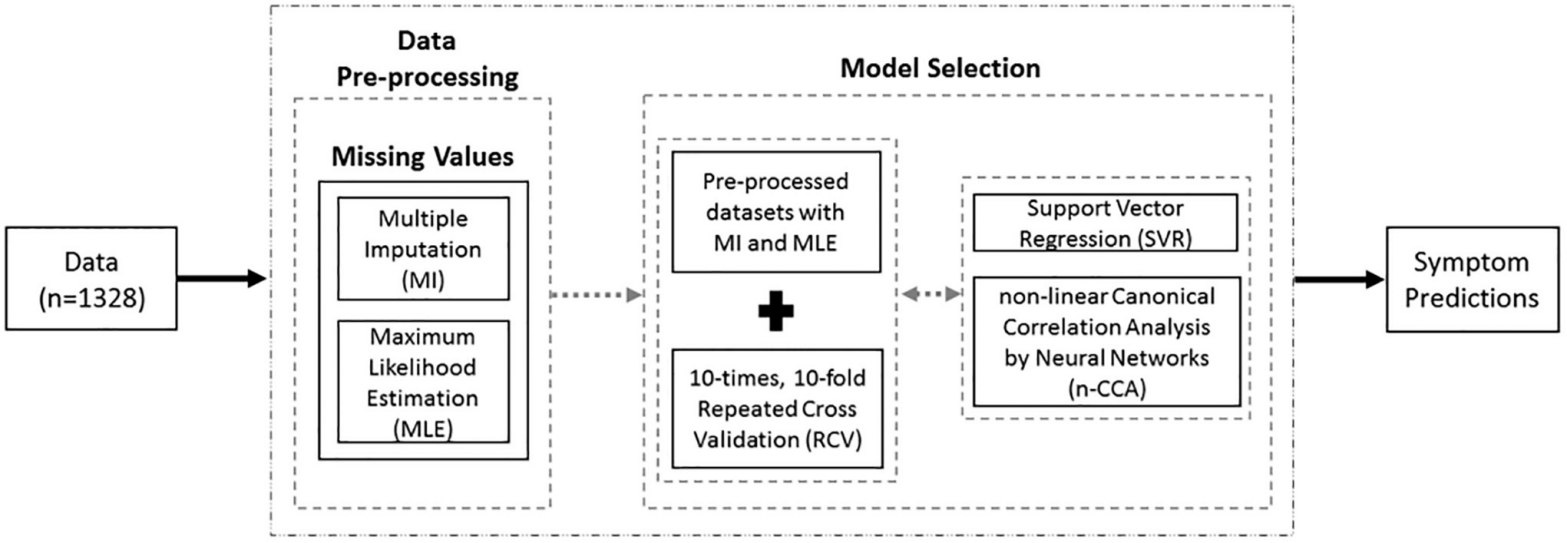
Overview of our analytic approach to learn from data to predict future symptoms of oncology patients.

A large variety of machine learning techniques and algorithms can be used to predict data by learning from previous observations. Choosing the most appropriate one for the prediction of symptom severity is a challenging task. Several common problems exist with this type of research including: small sample sizes; a significant number of missing values; the large number of symptom assessment instruments with different measurement scales; the different types of variables (e.g., categorical, ordinal, continuous); and the subjective nature of symptom measurements, themselves. Regression analysis is a common supervised machine learning method that can be used to solve several biological and clinical problems. It is used to estimate the relationship between a dependent variable (i.e., depression, anxiety, sleep disturbance) and one or more independent variables (i.e., predictor(s)). Canonical Correlation Analysis is another analytical method for exploring the relationships between two multivariate sets of variables (e.g., set of variables from Time Point 1 (TP_1_) and Time Point 2 (TP_2_) of a chemotherapry (CTX) cycle). In this study, we used Support Vector Regression (SVR) with different kernels (i.e., linear, polynomial, radial sigma) and Non-linear Canonical Correlation Analysis by Neural Networks (n-CCA) [9] to predict efficiently our dependent variables (i.e., symptom severity scores of depression, anxiety and sleep disturbance at TP_2_). The Multiple Imputation (MI) and Maximum Likelihood Estimation (MLE) methods were applied in order to account for missing data. Similarly, in order to accommodate the small sample size and avoid over-training, we applied a 10-times Repeated 10-fold Cross-validation (RCV) to our predictive models. To the best of our knowledge, this study is the first of its kind in oncology symptom management to applying n-CCA to predict the severity of three common symptoms in oncology patients. An overview of our analysis is provided at Fig 1.

Our study is organized as follows: the Methods section provides the research methodology along with all of the approaches used in the proposed model. The Results section presents the comparison and evaluation of the aforementioned methods and provides a summary of our results.

## Materials and methods

### Study procedure

The study, from which our data was drawn, was approved by the Committee on Human Research at the University of California, San Francisco and by the Institutional Review Board at each of the study sites. From February 2010 to December 2013, all eligible patients were approached by the research staff in the infusion unit to discuss participation in the study. Written informed consent was obtained from all patients. Depending on the length of their CTX cycles, patients completed questionnaires in their homes, a total of six times over 2 cycles of CTX (i.e. prior to CTX administration (Time 1 and 4), approximately 1 week after CTX administration (Time 2 and 5), approximately 2 weeks after CTX administration (Time 3 and 6). For this study, which is a secondary analysis of existing data, symptom data from the Time 1 and Time 2 assessment were analysed. Patients were asked to report on their symptom experience for the previous week. Medical records were reviewed for disease and treatment information. The methods for the parent study are described in fully detail in previously published work. [2, 10, 11]

### Patients and settings

We carried out a secondary analysis of existing data from this longitudinal study of the symptom experience of oncology outpatients receiving CTX. The data used in this study were obtained from the same dataset and relate to two different Time Points (i.e., Time Point 1 (TP_1_, n_1_ = 1343; prior to CTX administration), Time Point 2 (TP_2_, n_2_ = 1278; one week after CTX administration).

According to the study’s eligibility criteria: patients were ≥ 18 years of age; had a diagnosis of breast, gastrointestinal (GI), gynecological (GYN), or lung cancer; had received CTX within the preceding four weeks; were scheduled to receive at least two additional cycles of CTX; were able to read, write, and understand English; and gave written informed consent. Patients were recruited from two Comprehensive Cancer Centers, one Veteran’s Affairs hospital, and four community-based oncology programs.

### Instruments

The study instruments included a demographic questionnaire, the Karnofsky Performance Status (KPS) scale, [12, 13] the Self-administered Comorbidity Questionnaire (SCQ), [14] the Lee Fatigue Scale (LFS), [15] the Attentional Function Index (AFI), [16, 17] the General Sleep Disturbance Scale (GSDS), [18] the Center for Epidemiological Studies-Depression Scale (CES-D), [19] and the Spielberg State-Trait Anxiety Inventories (STAI-S and STAI-T). [20]

The demographic questionnaire provided information on age, marital status, years of education, living arrangements, ethnicity, employment status and exercise. In addition patients’ medical records were reviewed to obtain information on: body mass index (BMI), hemoglobin (Hgb), type of cancer, number of metastatic sites, time since cancer diagnosis, number or prior cancer treatments, and CTX cycle length.

To estimate changes in self-reported sleep disturbance, the GSDS was administered at each time point. The GSDS consists of 21 items designed to assess the quality of sleep in the past week. Each item was rated on a 0 (never) to 7 (every day) numeric rating scale (NRS). The GSDS total score is the sum of the 21 items that can range from 0 (no disturbance) to 147 (extreme sleep disturbance). A GSDS total score of ≥ 43 indicates a significant level of sleep disturbance. [21] The GSDS has well-established validity and reliability in shift workers, pregnant women, and patients with cancer and HIV. [18, 22, 23]

The CES-D consists of 20 items selected to represent the major symptoms in the clinical syndrome of depression. Scores can range from 0 to 60, with scores ≥ 16 indicating the need for individuals to seek clinical evaluation for major depression. The CES-D has well-established concurrent and construct validity. [19, 24, 25]

The STAI-T and STAI-S inventories consist of 20 items each that are rated from 1 to 4. The scores for each scale are summed and can range from 20 to 80. A higher score indicates greater anxiety. The STAI-T measures an individual’s predisposition to anxiety determined by his/her personality and estimates how a person feels generally. The STAI-S measures an individual’s transitory emotional response to a stressful situation. It evaluates the emotional response of worry, nervousness, tension, and feelings of apprehension related to how people feel “right now” in a stressful situation. The STAI-S and STAI-T inventories have well-established criteria and construct validity and internal consistency reliability coefficients. [20, 26, 27]

### Data analysis and missing data

Our data were collected from a cohort of oncology patients at two different Time Points, Time Point 1 (i.e., TP_1_, n_TP1_ = 1343), Time Point 2 (i.e., TP_2_, n_TP2_ = 1278). By merging the two different Time Points we created a new dataset of 1278 samples (n_TP1+TP2_ = 1278). When we dropped the cases with at least one missing value in one of their variables, we were left with 799 cases (65,1% of n_TP1+TP2_). To assess whether the missing values were missing completely at random (MCAR), missing at random (MAR), or missing not at random (MNAR) [28, 29] we analysed our data with SPSS version 23 (IBM, Armonk, NY). Furthermore, in order to avoid the problem of biasing our analysis by including only the complete cases, we used two different statistical approaches to impute the missing values, namely, Multiple Imputation (MI) [29, 30] and the Maximum Likelihood Estimation (MLE) [29, 31].

**Fig 2:**
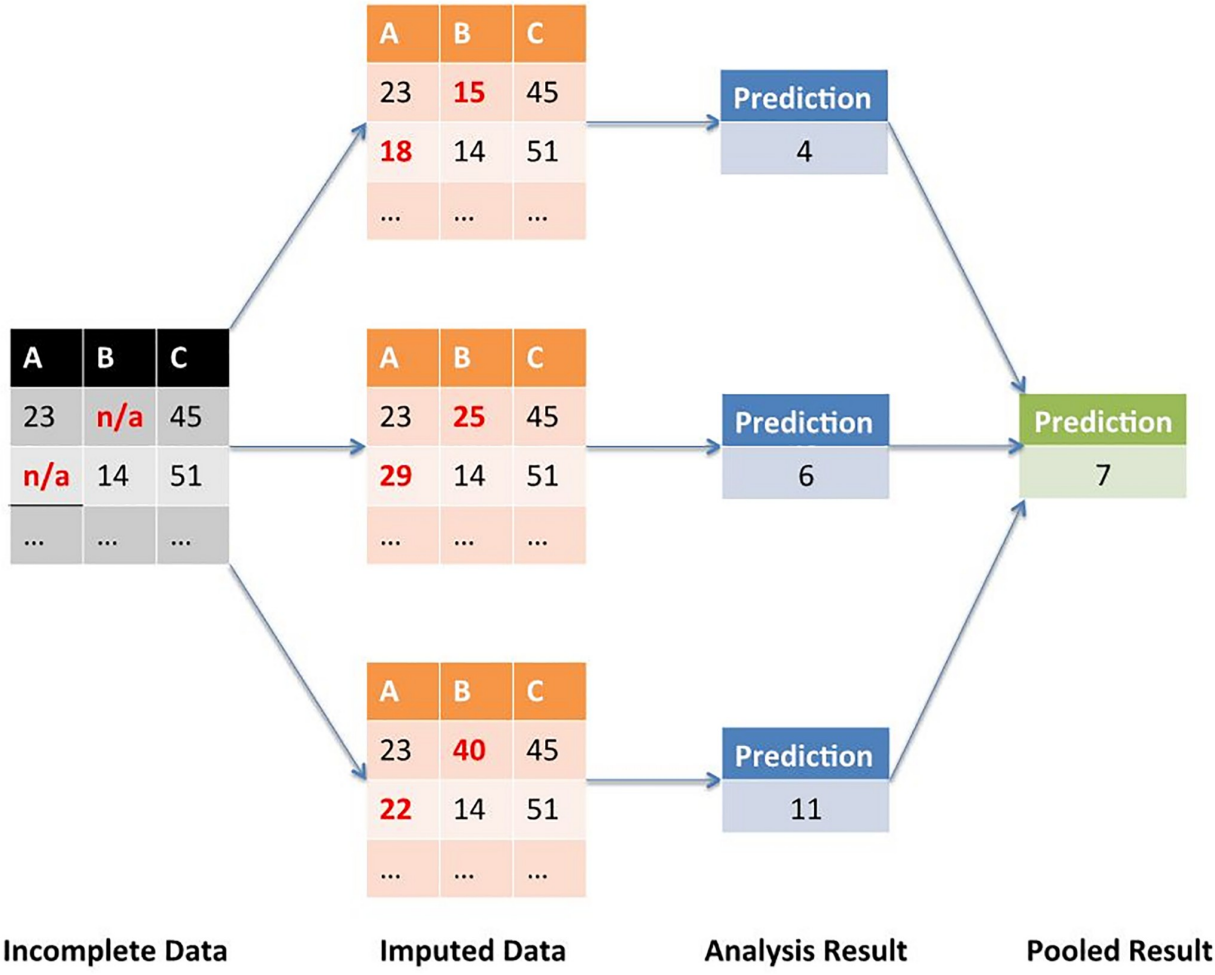
Multiple imputation.

Multiple Imputation (MI) is a statistical approach to address the problem of the missing observations that are frequently encountered in all types of epidemiological and clinical studies. [32] It minimizes the uncertainty around our missing data by creating different imputed data sets several times and integrating their results into a final, pooled result. Fig 2 illustrates the MI procedure with an example.

During the first stage, MI creates multiple copies of the dataset, with the missing values replaced by imputed values. These are sampled from their predictive distribution based on the observed data. [30] MI must fully account for all uncertainty in predicting the missing values by inserting appropriate variability into the multiple imputed values. During the second stage, MI fits the model of interest to each of the imputed datasets. The predicted estimations in each of the imputed datasets will differ because of the variation introduced in the imputation of the missing values. These estimates are only useful when averaged together to give the overall, pooled predicted associations.

Maximum Likelihood Estimation (MLE) is a common statistical method for estimating the parameters of a specific model, by finding the parameter values that maximize the likelihood of making the observations given these parameters. To elaborate more on this topic, [29] suppose we try an experiment with N people where the probability of a success for an individual is p and the probability of a failure is 1-p. If n people succeed and N-n people fail, the likelihood is proportional to the product of the probabilities of successes and failures or *p*^*n*^ × (1 − *p*)^*N* − *n*^. The value of p that maximizes the likelihood is n/N or the overall proportion of success. In our analysis for example, maximum likelihood produces the best estimate of the difference in the parameters between TP_1_ and TP_2_ that maximize the probability of observing the collected data. Unlike MI, MLE provides a unique estimate of the missing values and it requires fewer decisions than MI.

To impute the missing values with the MI approach and the MLE, we used SPSS version 23 (IBM, Armonk, NY). For the MI approach, we configured SPSS to automatically choose an imputation method based on a scan of our data and produce 10 output datasets with imputed values. For the MLE approach, we configured SPSS AMOS to use an independence model with a regression imputation and produce 1 output dataset with imputed values.

### Model selection

To train and evaluate the performance of our different predictive models we divided our dataset into two sub-sets: the Training (n_Train_ = 1000) and Validation (n_Val_ = 278) datasets. Cross-validation (CV) and Bootstrap are two common approaches that are known to provide unbiased estimates for the test results of a predictive model. [33] Cross-validation (CV) provides unbiased results but with a high error variance. On the other hand, Bootstrap is known to have better performance in small samples, achieving a small variance but requiring much heavier computation than CV. Combining the strengths of both approaches, Repeated Cross-validation (RCV) appears to be a good validation method for general use providing small bias with limited variability and a reasonable computation load. [33] In fact, RCV is a repeated CV method, in which the CV is repeated several times and then the average is taken. By the same rationale, Boostrap .632 is designed to address the pessimistic bias of the standard Bootstrap method, where the Bootstrap samples only contain approximately 63.2% of the unique samples from the original dataset. [34, 35]

Before training our models on the data, we empirically compared all the aforementioned validation methods on the original dataset before and after imputation for the missing values. Based on the validation method results, we compared the two different types of predictive models in our study with a 10-times and 10-fold RCV method.

We divided the original dataset with the missing values into a Training set of n_Train_ = 624 cases, and a Test set of n_Test_ = 175 cases. For the MI and MLE imputation methods, we divided the datasets into a Train set of n_Train_ = 1000 cases, and a Test set of n_Test_ = 278 cases. As already mentioned, the MI produced 10 such datasets, with each one of them having a total of n = 1278 cases.

#### Support Vector Regression

Support Vector Machine (SVM) is a popular machine learning algorithm used to analyze a variety of oncology data, [36–38] among many other applications. SVM became increasingly popular because of its successful application for a different set of problems (e.g. image recognition, text categorization, biosignals, bioinformatics). [39–41] SVM works by mapping data to a high-dimensional feature space so that data points can be categorized, even when the data are not otherwise linearly separable. SVM manages this challenge with an operation called the kernel trick. Through a variety of different kernel functions (e.g. Linear, Polynomial, Radial Basis Function), SVM takes low dimensional input space and transforms it to a higher dimensional space, thus converting non-separable problems to separable ones. With SVM, the data are transformed in such a way that separators between the different categories of the dataset can be found, optimized, and drawn. These separators are called the Optimal Separation Hyperplanes (OSH).

**Fig 3:**
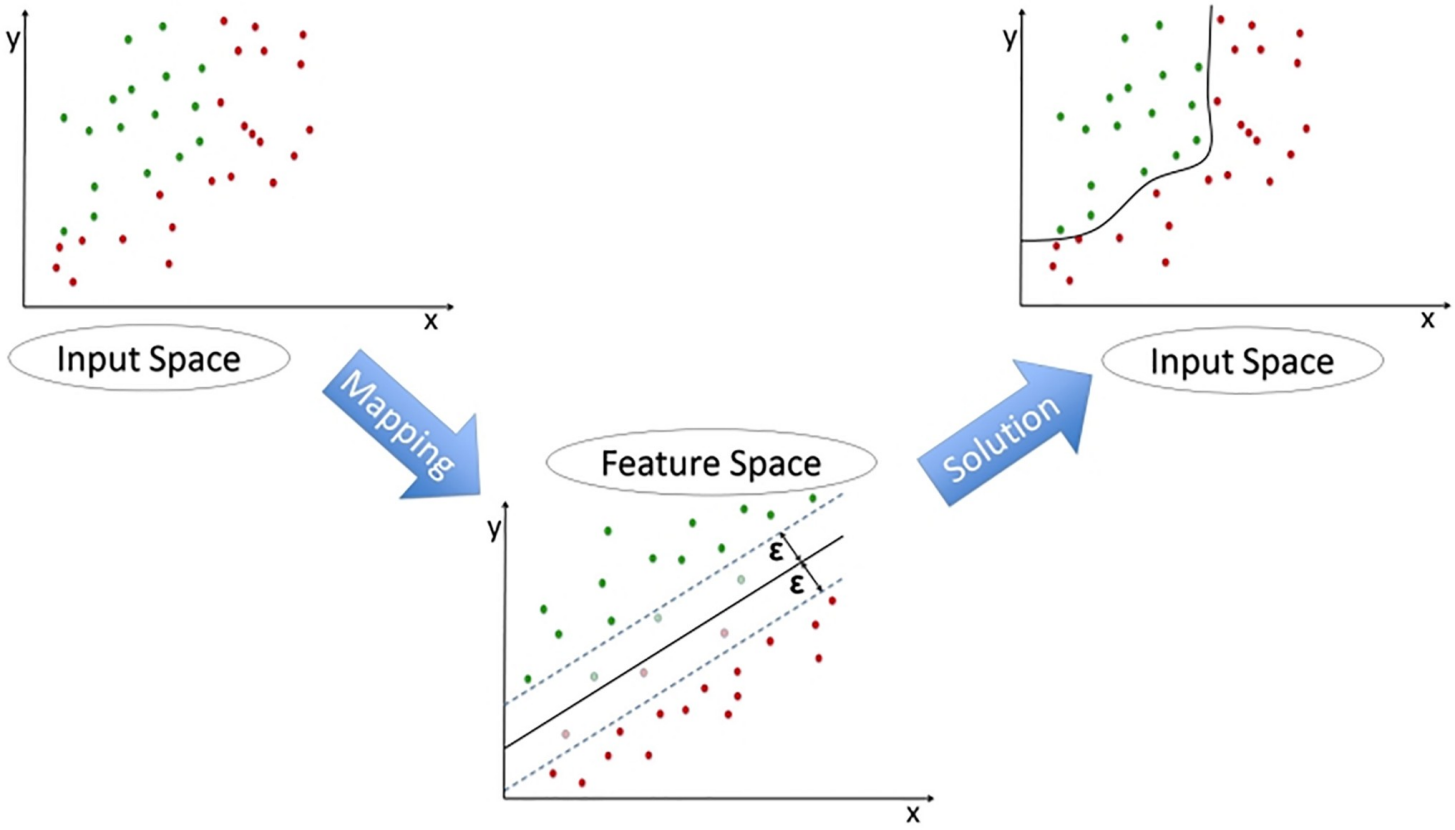
Support Vector Regression.

Support Vector Regression (SVR) is an extension of the SVM classifier, estimating the continuous function of a specific dataset. [42, 43] Similarly to SVM, SVR can model complex non-linear relationships by using an appropriate kernel function which maps the input data points onto a higher-dimensional feature space, transforming the non-linear relationships into linear forms. The efficiency of the procedure is determined by the kernel function’s parameters which do not depend on the dimensionality of feature space. Both SVM and SVR depend on defining a loss function, called epsilon intensive (*ϵ*), which ignores the errors that are situated within a certain distance of the true value. Fig 3 shows an example of a non-linear regression function with its epsilon intensive band. In our study, we implemented all the different SVR models using R version 3.3.0 and the Caret Package. [44]

#### Non-linear Canonical Correlation Analysis by neural networks

In our study, we adapted the n-CCA which was introduced by Hsieh et al. in 2000. [9] Our implementation was done with PyCharm Professional Edition 4.5, using Python 2.7 and the Scikit-Learn, Theano, and Lasagne libraries. [45–47]

Canonical Correlation Analysis (CCA) is a method to identify the linear combinations of a set of variables X that have the highest correlations with linear combinations of a set of variables Y. It estimates the correlated modes between the two data sets of variables X (i.e, the data from TP_1_) and Y (i.e., the data from TP_2_) by solving the equations,

$$\begin{array}{c} i)\;U=a*X,\;ii)\;V=b*Y, \end{array}$$ (1)

while maximising the Pearson correlation between U and V. To achieve a better performance in cases where the correlation between the two data sets is non-linear, the equations can be modified to include a non-linear relationship. Hsieh et al. [9] have introduced an implementation of n-CCA utilising three neural networks. For a more in-depth mathematical description of the method, we refer to the original paper. [9] In this paper, we describe the concepts of how the neural networks can be used to extract the correlation between the two sets of variables (i.e., data from TP_1_ and TP_2_).

**Fig 4:**
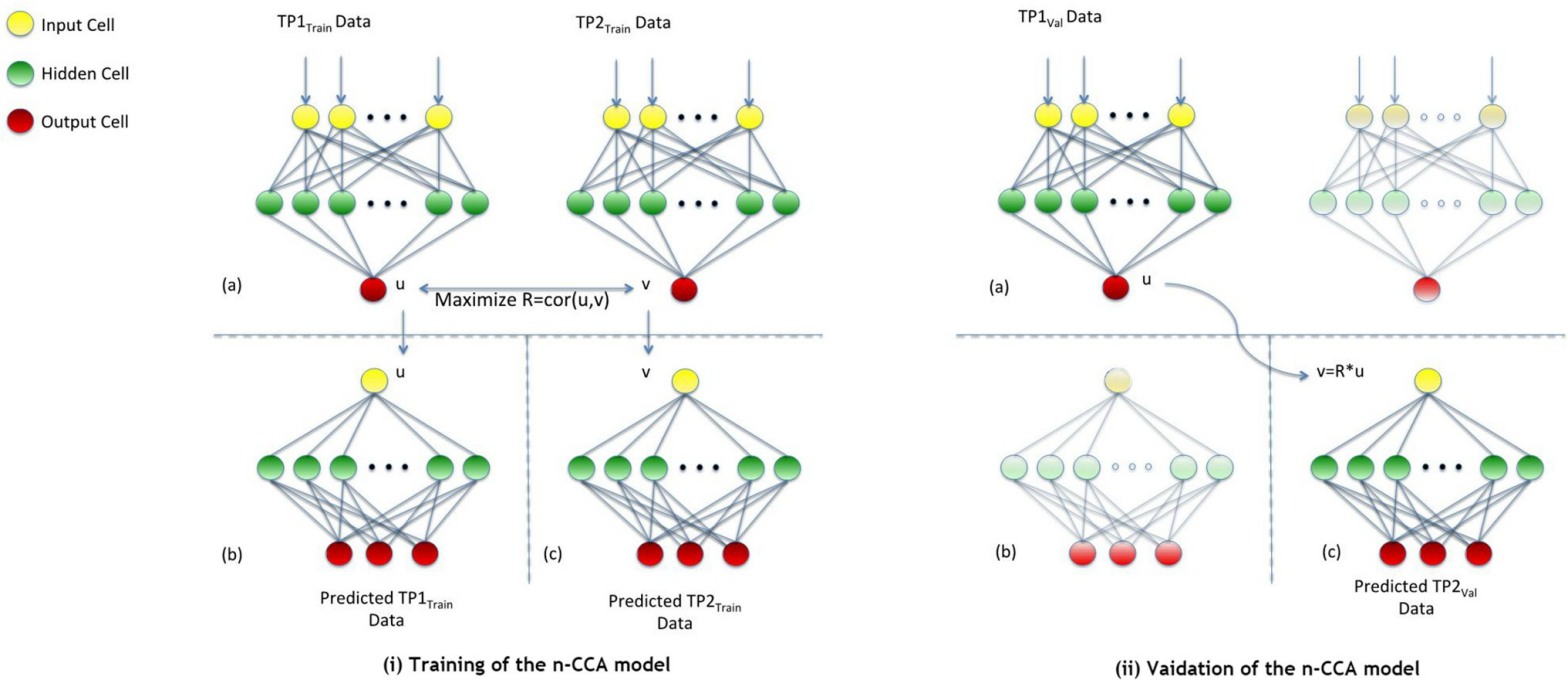
n-CCA training and validation: (i) training of the n-CCA model, (ii) validation of the n-CCA model.

Fig 4 shows the architecture, as well as the training and validation stage, of our neural network that implements n-CCA with our data from TP_1_ and TP_2_. Our model consists of three networks which were trained separately. The first neural network is a double-barrelled one (illustrated in the Training-(a) section of Fig 4). We called this network the inner network and the other two the outer networks (shown in the Training-(b) and Training-(c) sections of Fig 4).

The two barrels of the inner network (Training-(a) section of Fig 4) share the same structure and take as input the data from TP_1_ and TP_2_ respectively. The input layer of each barrel has n nodes, one for each feature of the data (n_Features_ = 29); see S1 Table for a full list of these features). The hidden layer of each barrel contains 50 nodes, that have the hyperbolic tangent function as their activation function. The output layer of each barrel has only one node, producing an output U for the dataset from TP_1_ and an output V for the data set from TP_2_ respectively. The aim of the training of the inner network is to maximise the correlation between the two vectors, U and V. This aim is achieved by using the negative Pearson coefficient [48] as the cost function which has to be minimised.

The outer networks (Training-(b) and Training-(c) sections of Fig 4) are trained separately, nevertheless they share the same structure. The input layer of each outer network has only one node, taking as input the output (U and V respectively) of the previous double-barrel inner network. The hidden layer of each of the outer networks has 50 nodes with the hyperbolic tangent function as their activation function. The output layer of each outer network produces the features that we need to predict. In our study, we predicted the severity of three symptoms (i.e., sleep disturbance, depression, anxiety). To predict them, each outer network learns the inverse function of each of the barrels of the inner network and maps U and V, respectively, back to the features we are interested to predict. The first outer network (Training-(b) section of Fig 4) maps U back to predicted values of sleep disturbance, depression and anxiety for TP_1_ and the second outer network (Training-(c) section of Fig 4) maps V back to predicted values of sleep disturbance, depression and anxiety for TP_2_. The aim of the training of the outer networks is to minimise the Mean Squared Error (MSE) between the predicted output and the true values. This training phase consists of 100 epochs, during which we used a 10-times and 10-fold RCV.

When the training stage (Training section of Fig 4) is finished, we can use parts of the model to predict TP_2_ data from new, unseen TP_1_ data (Validation section of Fig 4). This process can be used either to validate the model or to predict the TP_2_ data, when new patients are introduced into the model, where only the TP_1_ data are available. In both cases, the TP_1_ data are fed into the left barrel of the inner network (Validation-(a) section of Fig 4) to estimate the U vector for these data. By multiplying this output with the Pearson coefficient R that was calculated during the training stage, we can estimate the corresponding V vector. This information forms the input for the outer right network (Validation-(c) section of Fig 4), which predicts the desired features for the TP_2_ data.

### Comparison between SVR and n-CCA

To compare the performances of the SVR and n-CCA models we used their Root Mean Square Error (RMSE) and Normalised Root Mean Square Error (NRMSE). The latter, was calculated by dividing their RMSE with the mean of the measured values.

$$\begin{array}{c} NRMSE=\frac{RMSE}{mean}, \end{array}$$ (2)

Normalising the RMSE allows the comparison of models with different scales. Lower values among these calculations indicate less residual variance for the predicted outcomes.

**Fig 5:**
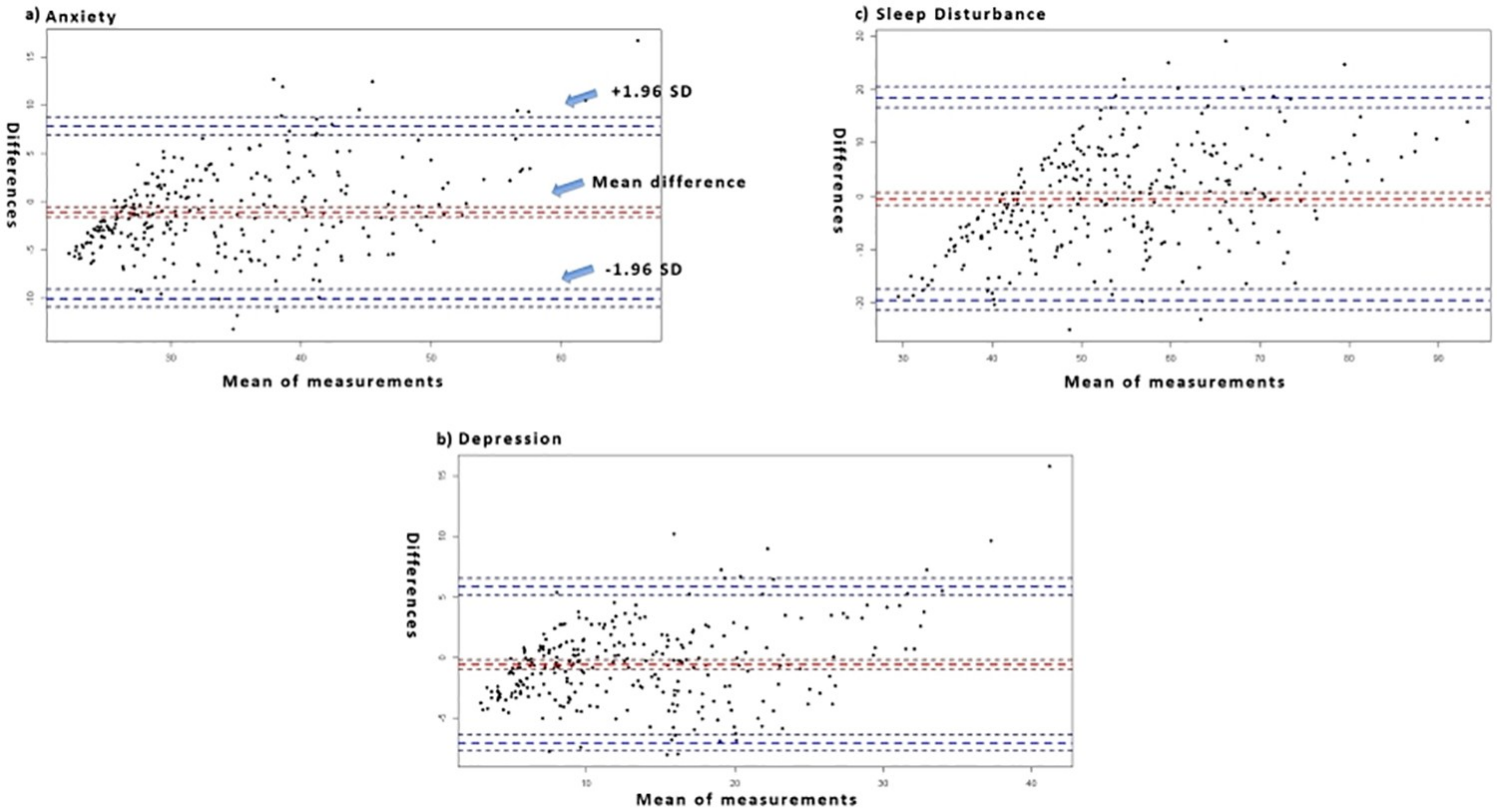
Bland—Atman plot of the SVR model with the polynomial function and the n-CCA model on the dataset with Maximum Likelihood imputation.

In order to compare the results produced with the SVR and the n-CCA models we used the Bland–Altman plot. [49, 50] The Bland-Altman plot is a graphical method to compare two different measurement techniques. The difference between each technique’s measurement for each case is plotted against the average of the other technique’s measurement for the same case. The former is represented on the y-axis and the latter on the x-axis. The full Bland-Altman plot draws these differences and averages for every case in the test dataset. In our study, the mean difference and the mean difference plus and minus 1.96 times the standard deviation of the differences are represented on the Bland-Altman plot with horizontal lines (see Fig 5).

In order to evaluate the agreement between the real and predicted values, we compared the mean, range, and kernel density plots of the results from the analyses on the dataset with the MLE imputation. We compared the predictions of the SVR with polynomial kernel and n-CCA models against the real values of our Test set (n_Test_ = 278 cases).

## Results

### Data analysis and handling missing data

**Fig 6:**
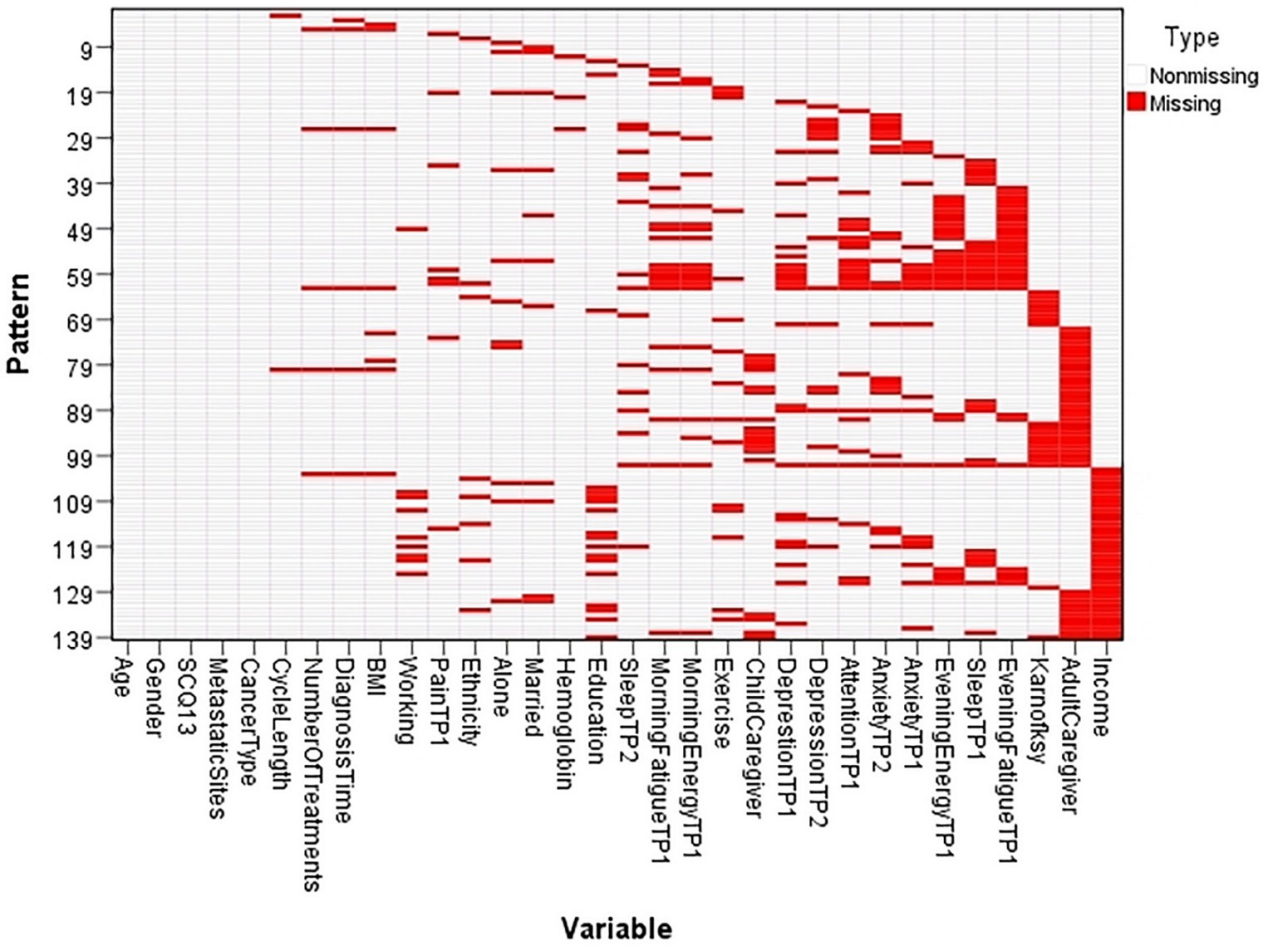
Missing values pattern (Little’s MCAR test, p>0.05).

Our initial dataset that contained the data from both TP_1_ (prior to CTX administration) and TP_2_ (one week after CTX administration; n_TP1+TP2_ = 1278), had 799 fully completed cases (65,1% of n_TP1+TP2_). The empty values in the dataset were missing completely at random (Little’s MCAR test, p>0.05; Fig 6). These missing values are due to missing responses from patients. In order to use the collected values of all of our cases, we applied the MI [29, 30] and MLE [29, 31] to compensate for missing values.

### Model selection

**Fig 7:**
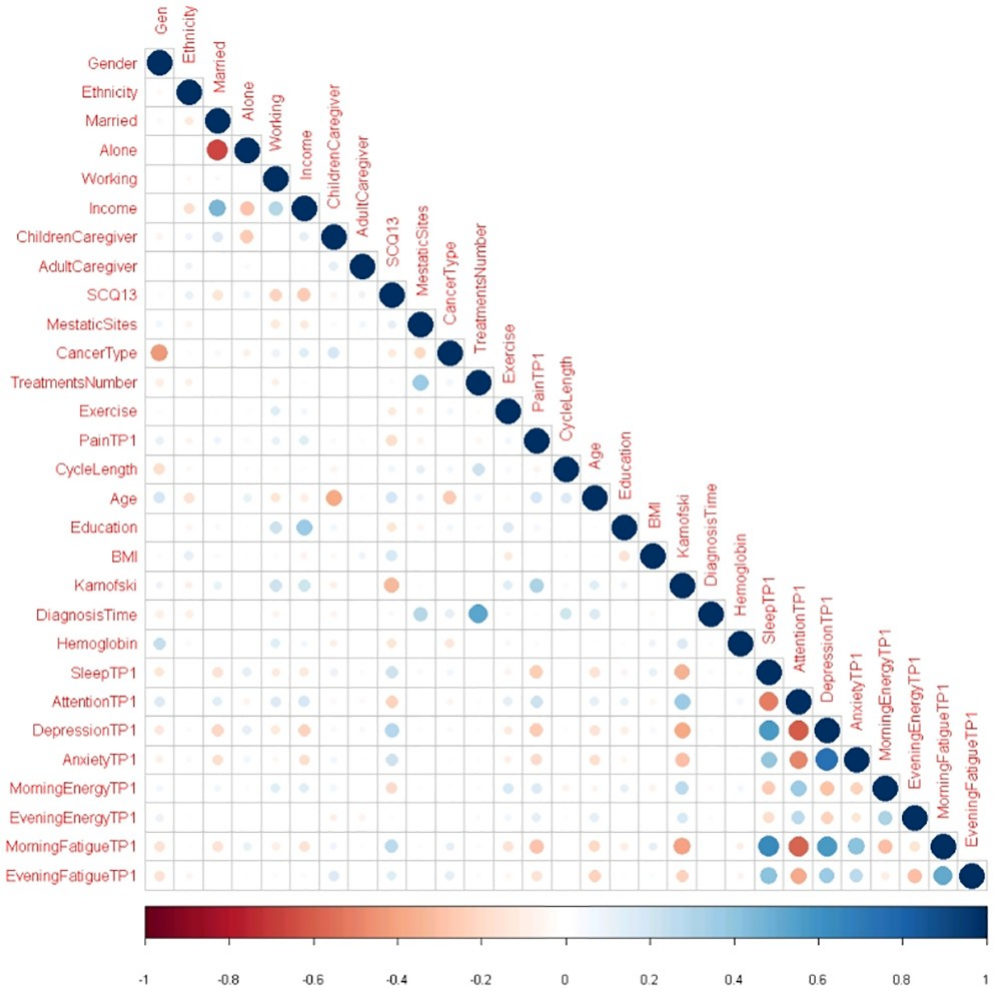
Correlation analysis of predictor variables.

As our validation method, we selected a 10-times and 10-fold RCV. Beforehand, we compared the performance of this validation method with Bootstrap, Bootstrap .632 and 10-fold CV. As predictor variables we used the data collected from TP_1_ (for a full description of these predictors see S1 Table). Moderate correlations were found among a number of predictors (see Fig 7). Type of cancer was correlated with gender because 40.6% (n = 519) of the patients in our study had breast cancer. The number of prior cancer treatments was correlated with time from patients’ initial cancer diagnosis. Income was correlated with being married and living alone. KPS score [12, 13] was moderately correlated with sleep disturbance, attentional function, depression, and morning fatigue. Finally regarding the symptoms collected in our dataset, moderate correlations were found between sleep disturbance, anxiety, depression, attentional function, morning energy, and morning fatigue.

Our analytical models were implemented in all three types of datasets (i.e. the original ones with the missing values, the ones imputed with MI, the ones imputed with MLE). The performance of the models was evaluated based in the RMSE and the R-squared (R^2^). Table 1 shows the performance of different SVR models for predicting depression (CES-D) at TP_2_.

**Table 1:** Performance of Support Vector Regression (SVR) models for predicting depression (CES-D) at TP_2_.

		10-times Repeated 10-fold CV		10-fold CV		Bootstrap		Bootstrap .632	
Dataset	Kernel	RMSE	R^2^	RMSE	R^2^	RMSE	R^2^	RMSE	R^2^
**Missing data**	Linear	6.484	0.589	6.484	0.589	6.484	0.589	6.484	0.589
	Polynomial	6.435	0.592	6.435	0.592	6.436	0.592	8.268	0.416
	Radial Sigma	6.475	0.591	6.470	0.592	6.473	0.591	6.752	0.559
**Multiple Imputation**	Linear	6.201	0.644	6.201	0.644	6.201	0.644	6.201	0.644
	Polynomial	6.191	0.644	6.191	0.644	6.193	0.644	8.121	0.517
	Radial Sigma	6.401	0.628	6.387	0.630	6.389	0.630	6.512	0.587
**Maximum Likelihood**	Linear	7.102	0.548	7.102	0.548	7.102	0.548	7.102	0.548
	Polynomial	7.081	0.549	7.081	0.549	7.053	0.552	9.954	0.323
	Radial Sigma	7.189	0.540	7.182	0.541	7.192	0.540	7.393	0.508

Based on these results (Table 1), we selected the 10-times and 10-fold RCV as our preferred validation method to test and compare the remaining analyses. The best overall performance for predicting CES-D at TP_2_ with SVR was implemented with the polynomial kernel on the MI imputed dataset (RMSE = 6.191, R^2^ = 0.644). This result could be due to the method of imputation used in the latter dataset and the type of kernel function that was used to construct the prediction model. All four validation methods provided equivalent results with their best performance implemented on the MI datasets. Bootstrap .632 combined with the polynomial kernel had the worst performance on all three types of datasets (i.e. RMSE = 9.954, R^2^ = 0.323).

### Comparison between SVR and n-CCA

To compare the different approaches used in our study, we applied three different SVR and three n-CCA models to predict the severity of depression (CES-D), sleep disturbance (GSDS), and state anxiety (STAI-S) at TP_2_. The SVR models were implemented with three different kernels (i.e. Linear, Polynomial, Radial Sigma). All of our models were tested on all three types of datasets. As predictors we used all the data collected at TP_1_ (see S1 Table).

We compared the performance of the models based on their RMSE, their RMSE/mean ratio (see Tables 2 & 3), and their differences in the Bland-Atman plots (Fig 5). In general the SVR models provided better fitted models with lower prediction error. All the models provided better results using the MI dataset. For the prediction of sleep disturbance, the SVR with the polynomial kernel achieved a RMSE of 13.153 and a RMSE/mean ratio of 0.209. For sleep disturbance, the n-CCA achieved a RMSE of 16.113 and R^2^ of 0.306. For the prediction of anxiety, the polynomial kernel achieved a RMSE of 7.983 and a RMSE/mean ratio of 0.220. For anxiety the n-CCA achieved a RMSE of 8.941 and a RMSE/mean ratio of 0.677. Finally, for the prediction of depression, the polynomial kernel achieved a RMSE of 6.191 and a RMSE/mean ratio of 0.465. For depression, the n-CCA achieved a RMSE of 6.907 and a RMSE/mean ratio of 0.221.

**Table 2:** Performance of Support Vector Regression (SVR) models for predicting sleep disturbance (GSDS), anxiety (STAI-S) and depression (CES-D) at TP_2_.

		Sleep Disturbance		Anxiety		Depression	
Dataset	Kernel	RMSE	RMSE/mean	RMSE	RMSE/mean	RMSE	RMSE/mean
**Missing data**	Linear	13.302	0.251	8.084	0.244	6.484	0.509
	Polynomial	13.379	0.251	8.082	0.245	6.435	0.506
	Radial Sigma	13.709	0.258	8.147	0.247	6.475	0.518
**Multiple Imputation**	Linear	13.156	0.212	7.985	0.221	6.201	0.465
	Polynomial	13.153	0.209	7.982	0.220	6.191	0.465
	Radial Sigma	13.243	0.239	8.045	0.228	6.401	0.488
**Maximum Likelihood**	Linear	13.316	0.248	8.583	0.256	7.102	0.537
	Polynomial	13.331	0.246	8.476	0.251	7.081	0.536
	Radial Sigma	13.836	0.256	8.625	0.258	7.189	0.556

**Table 3:** Performance of n-CCA for predicting sleep disturbance (GSDS), anxiety (STAI-S) and depression (CES-D) at TP_2_.

	Sleep Disturbance		Anxiety		Depression	
Dataset	RMSE	RMSE/mean	RMSE	RMSE/mean	RMSE	RMSE/mean
Missing data	19.955	0.307	12.238	0.681	9.661	0.222
Multiple Imputation	16.113	0.306	8.941	0.677	6.907	0.221
Maximum Likelihood	16.680	0.305	9.320	0.676	7.583	0.218

Regarding the discrepancies between the two types of measurements as shown on the Bland-Atman plots (Fig 5), the mean differences in the measurements of all three symptoms (i.e., sleep disturbance, anxiety, depression) were close to zero. Most of these differences were between +1.96SD and -1.96SD from the mean difference, which suggests a normal distribution. The two types of analysis (i.e., SVR with polynomial kernel and n-CCA) show a moderate to high level of agreement between their measurements.

#### Comparison between the real and predicted values

**Fig 8:**
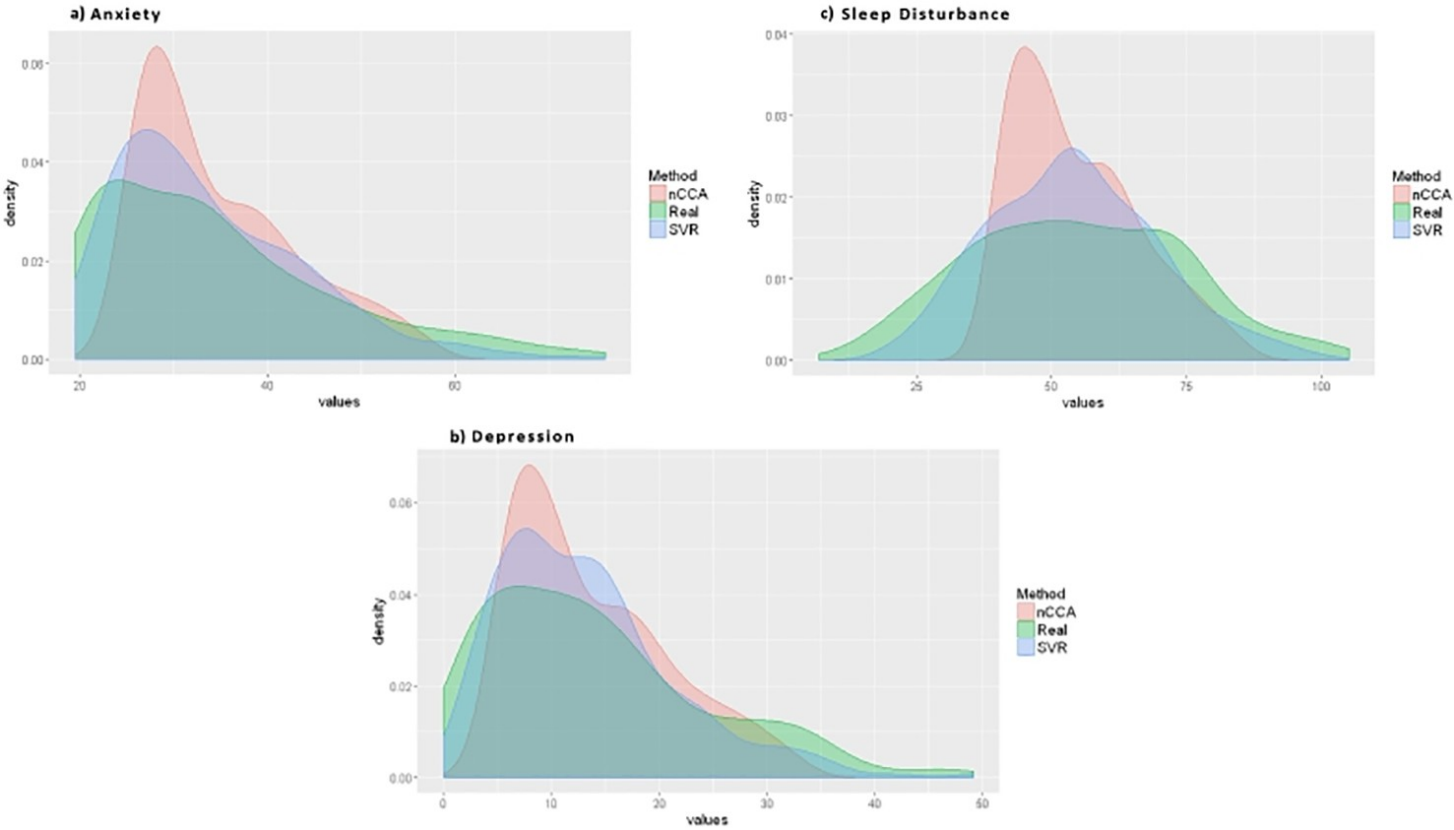
Density plots of the sleep disturbance (GSDS), anxiety (STAI-S) and depression (CES-D) real values compared to the density plots of predicted values with the SVR (polynomial kernel) and n-CCA on the dataset with the Maximum Likelihood Estimation imputation.

For all three symptoms (i.e., sleep disturbance, anxiety, depression), the means of the predicted values were very close to the means of the real values (Table 4). Regarding their ranges, the ranges of the predicted values from the SVR models were much closer to the ranges for the real values (Table 4). In general, the distributions of predicted values, from both analytical models, were very similar to the distributions for real values (Fig 8). n-CCA, as a Neural Network based algorithm, appears to be affected by our relatively small sample size and the distribution of data on the edges of the symptom scales. In general, it performed better where the data were denser (i.e., more data). On the other hand, SVR with polynomial kernel appears to be less affected by the aforementioned factors and provided predicted values with a high concordance with the real values.

**Table 4:** Sleep disturbance (GSDS), anxiety (STAI-S) and depression (CES-D) real values compared to the predicted values with the SVR (polynomial kernel) and n-CCA on the dataset with the Maximum Likelihood Estimation imputation.

Symptoms	Real Values (mean)	Real Values (range)	SVR (polynomial kernel)				n-CCA			
			Predicted Values (mean)	Predicted Values (range)	RMSE	RMSE / mean	Predicted Values (mean)	Predicted Values (range)	RMSE	RMSE / mean
**Sleep Disturbance**	54.796	7.000 - 105.000	54.089	20.044 - 100.214	13.331	0.246	54.600	38.427 - 86.368	16.680	0.305
**Anxiety**	34.481	20.000 - 76.000	33.749	19.495 - 74.236	8.476	0.251	34.865	24.895 - 57.583	9.320	0.267
**Depression**	14.119	0.000 - 49.000	13.205	0.097 - 49.110	7.081	0.536	13.792	4.578 - 33.338	7.583	0.550

## Conclusion

This study is the first to use two different machine learning techniques to accurately predict the severity of three common symptoms (i.e., sleep disturbance, anxiety, depression) from prior to through one week following the administration of CTX. The predictions were constructed using the features of the experimental dataset collected at the first Time Point. Using the SVR method, the differences between the real values (i.e., symptom severity scores that the patients reported) and the predicted values were not meaningful differences. Furthermore, we obtained fairly similar results with n-CCA at the expense of having a smaller variance among the predicted values (i.e. higher ratio of RMSE/mean in most cases). The results indicate that relatively similar findings were obtained independent of the number of missing values or the imputation method used to compensate for missing values in our dataset. The ability to predict the severity of future symptoms in oncology patients will be a powerful tool for oncology clinicians. Developing computational tools using machine learning techniques will assist clinicians to risk profile patients and implement pre-emptive symptom management interventions. Using this information, clinicians will be able to customize a patient’s treatment, increase their tolerance for CTX, and improve their quality of life. Following replication, these methods can be evaluated as a decision support tool to assist clinicians to improve symptom management in patients receiving CTX. Finally, the approaches presented in this paper, may be applicable to the same set of co-occurring symptoms in other chronic medical conditions.

The optimization of the feature selection process was one of the limitations of our study. Being an exploratory study for the performance of the aforementioned predictive models, we focused on the construction of predictive models and their evaluation and comparison. This effort was implemented through comparison of different imputation techniques (i.e. MI, MLE), validation (i.e. RCV, CV, Bootstrap, Bootstrap .632) and evaluation methods (i.e. RMSE, Bland-Altman plot). Future work will focus on defining an effective set of predictors, as well as pre-processing and enhancing the data collection and representation to improve the efficiency of both of the SVR and the n-CCA models. In addition, we will develop an incremental learning method with additional time points and evaluate it on a similar dataset. [51]

## Supporting information

S1 Table Predictor variables for the Support Vector Regression (SVR) and the Non-linear Canonical Correlation Analysis by neural networks models. (TIF)
