# Supplementary figures and images for: Learning from data to predict future symptoms of oncology patients
Source: PLoS One. 2018 Dec 31;13(12):e0208808. doi: 10.1371/journal.pone.0208808 (PMC6312306; doi:10.1371/journal.pone.0208808)

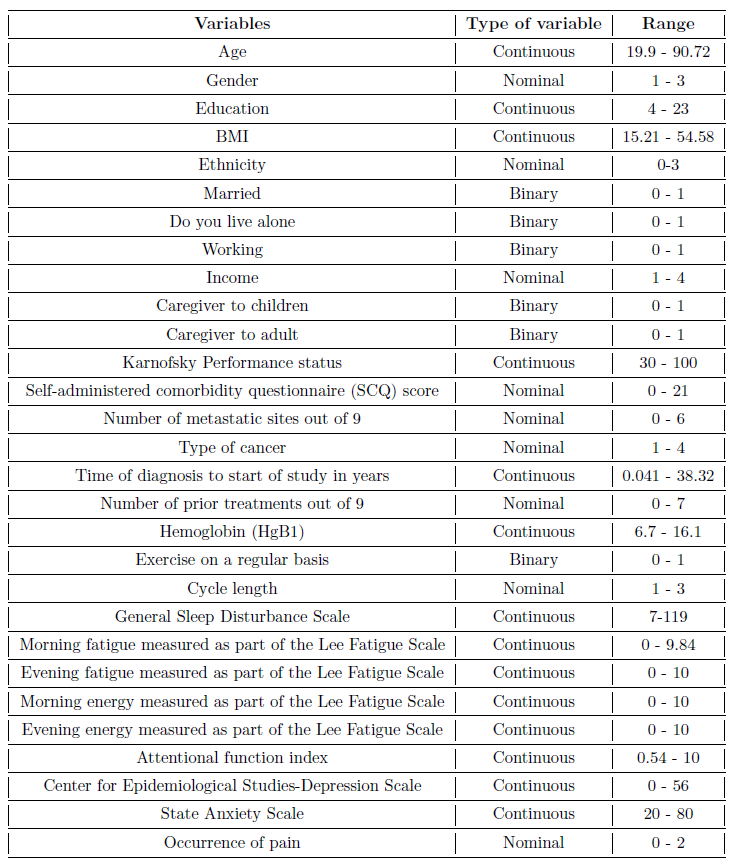

Supplement: S1 Table — (TIF) [file pone.0208808.s001.tif]
